# Neuroscience-based Nomenclature (NbN) and Early Career Psychiatrists: A Cross-Sectional Study on Views, Attainment and Needs

**DOI:** 10.1192/j.eurpsy.2024.1438

**Published:** 2024-08-27

**Authors:** A. Seker, D. Cavaleri, F. Santos Martins, S. Bianchi, D. Zani, S. Zemach, J. Zohar, A. Young

**Affiliations:** ^1^Child and Adolescent Psychiatry, Institute of Psychiatry, Psychology & Neuroscience, London, United Kingdom; ^2^Medicine and Surgery, University of Milano-Bicocca , Monza, Italy; ^3^Psychiatry, Centro Hospitalar Universitário S. João, Porto, Portugal; ^4^Psychiatry, University of Perugia, Perugia, Italy; ^5^Psychiatry, Psychiatric Services Grisons, Chur, Switzerland; ^6^Psychiatry, The Jerusalem Mental Health Center, Jerusalem; ^7^Chaim Sheba Medical Center, Tel Hashomer, Israel; ^8^Mood disorders, Institute of Psychiatry, Psychology & Neuroscience, London, United Kingdom

## Abstract

**Introduction:**

Anatomical Therapeutic Chemical (ATC) indication-based classification system is the World Health Organization (WHO) drug classification system and it is widely used in clinical and researh practice, however there has been questions around the scientific base of this (1, 2). Neuroscience-based Nomenclature (NbN) has been developed by representatives from 5 international organizations, with specific expertise in psychopharmacology, to address the issues around neuropsychopharmacological drug classification and improve the focus on pharmacological domains and mode of action:

**ECNP** – European College of Neuropsychopharmacology

**ACNP** – American College of Neuropsychopharmacology

**AsCNP** – Asian College of Neuropsychopharmacology

**CINP** – International College of Neuropsychopharmacology

**IUPHAR** – International Union of Basic and Clinical Pharmacology

References:

1. Nutt DJ. Beyond psychoanaleptics - can we improve antidepressant drug nomenclature? [published correction appears in J Psychopharmacol. 2009 Sept;23(7):861]. *J Psychopharmacol.* 2009;23(4):343-345. doi:10.1177/0269881109105498

2. Zohar J, Stahl S, Moller HJ, et al. A review of the current nomenclature for psychotropic agents and an introduction to the Neuroscience-based Nomenclature. *Eur Neuropsychopharmacol.* 2015;25(12):2318-2325. doi:10.1016/j.euroneuro.2015.08.019

**Objectives:**

As NbN is a novel classification system that can be used as a teaching tool as well as for other purposes, we aimed to understand the experience, views and needs of the psychiatric trainees and early career psychiatrists who will shape the future of psychiatry, around drug classification systems.

**Methods:**

The ethical clearance of the study was obtained from King’s College London. We prepared an online survey (https://forms.gle/FCSdVTFH4U5QNn5t8) with a multinational group of early career pscyhiatrists who met through the CINP and EFPT, and test-run the survey with a small group of psychiatric trainees. The online survey was then disseminated via emailing lists and groups of early careers psychiatrists as well as through social media.

**Results:**

At the time of this abstract submission, the data collection is ongoing. Results will include analyses of the experience with different drug classifcations systems, awareness, views and attainment of NbN, stratified according to the demographic data (country, careers status, main work setting).

**Conclusions:**

The findings from this study will shed light on the views and needs of early career psychiatrists on the topic from clinical and academic aspects, a previously unexplored perspective on drug classification systems. The findings can inform the planning of various strategies to address areas to improve the use and teaching of these tools.

**Disclosure of Interest:**

None Declared

